# Coordinated Regulation Among Progesterone, Prostaglandins, and EGF-Like Factors in Human Ovulatory Follicles

**DOI:** 10.1210/jc.2016-3153

**Published:** 2017-03-09

**Authors:** Yohan Choi, Kalin Wilson, Patrick R. Hannon, Katherine L. Rosewell, Mats Brännström, James W. Akin, Thomas E. Curry, Misung Jo

**Affiliations:** 1 Department of Obstetrics and Gynecology, University of Kentucky College of Medicine, Lexington, Kentucky 40536; 2 Department of Obstetrics and Gynecology, University of Gothenburg, 405 30 Gothenburg, Sweden; 3 Stockholm IVF, 112 81 Stockholm, Sweden; 4 Bluegrass Fertility Center, Lexington, Kentucky 40503

## Abstract

**Context:**

In animal models, the luteinizing hormone surge increases progesterone (P4) and progesterone receptor (PGR), prostaglandins (PTGs), and epidermal growth factor (EGF)–like factors that play essential roles in ovulation. However, little is known about the expression, regulation, and function of these key ovulatory mediators in humans.

**Objective:**

To determine when and how these key ovulatory mediators are induced after the luteinizing hormone surge in human ovaries.

**Design and Participants:**

Timed periovulatory follicles were obtained from cycling women. Granulosa/lutein cells were collected from *in vitro* fertilization patients.

**Main Outcome Measures:**

The *in vivo* and *in vitro* expression of PGR, PTG synthases and transporters, and EGF-like factors were examined at the level of messenger RNA and protein. PGR binding to specific genes was assessed. P4 and PTGs in conditioned media were measured.

**Results:**

*PGR*, *PTGS2*, and *AREG* expressions dramatically increased in ovulatory follicles at 12 to 18 hours after human chorionic gonadotropin (hCG). In human granulosa/lutein cell cultures, hCG increased P4 and PTG production and the expression of *PGR*, specific PTG synthases and transporters, and EGF-like factors, mimicking *in vivo* expression patterns. Inhibitors for P4/PGR and EGF-signaling pathways reduced hCG-induced increases in PTG production and the expression of EGF-like factors. PGR bound to the *PTGS2*, *PTGES*, and *SLCO2A1* genes.

**Conclusions:**

This report demonstrated the time-dependent induction of *PGR*, *AREG*, and *PTGS2* in human periovulatory follicles. *In vitro* studies indicated that collaborative actions of P4/PGR and EGF signaling are required for hCG-induced increases in PTG production and potentiation of EGF signaling in human periovulatory granulosa cells.

## Teaser

The LH surge increases prostaglandins and EGF-like factors by coordinated regulation by progesterone/PGR and EGF signaling in human periovulatory follicles.

The luteinizing hormone (LH) surge stimulates preovulatory follicles to produce local autocrine and paracrine mediators that coordinate complex intra- and extracellular events to bring about ovulation and luteinization. Such key local mediators include progesterone (P4) and its nuclear receptor [progesterone receptor (PGR)], prostaglandins (PTGs) (PGE_2_ and PGF_2_*_α_*), and epidermal growth factor (EGF)–like factors (AREG, EREG, and BTC). Despite abundant evidence that P4/PGR, EGFR signaling, and PTGs play a crucial role for successful ovulation in various animals models, little is known about exactly when and how the LH surge induces these mediators and how these mediators coordinate ovulatory changes during the periovulatory period in the human ovary. This is mainly because of extremely limited access to timed periovulatory follicles in the human ovary and the lack of well-established human granulosa cell models in which the cellular events induced by the LH surge *in vivo* can be recapitulated *in vitro*.

The LH surge or human chorionic gonadotropin (hCG) increases P4 production and *PGR* expression in periovulatory follicles, which is essential for successful ovulation in various animal models (1). For instance, blocking P4 biosynthesis (2) or obliterating the activity or expression of PGR by chemical inhibitors (3, 4) or gene deletion/silencing (5, 6), respectively, resulted in anovulation in various experimental animal models.

Various animal studies indicated that the LH surge or hCG induces rapid increases in the expression of *Areg*, *Ereg*, and *Btc* in periovulatory granulosa cells, and these factors act as key mediators of LH action in ovulatory follicles necessary for successful ovulation (7–9). Notably, mutant mice with compromised EGF signaling (*e.g.*, Ereg^−/−^* Egfr^wa2/ wa2^, Areg^−/−^* Egfr^wa2/wa2^) showed reduced cumulus oocyte complex (COC) expansion and ovulation (10).

The LH surge also increases the production of PTGs in periovulatory follicles, which is crucial for successful ovulation and COC expansion (11). For instance, *Ptgs2* and *Ptger2* knockout mice showed reduced ovulation rate and/or COC expansion (12, 13). In monkeys, follicular injection of PTG synthesis inhibitors prevented ovulation and cumulus expansion, which was restored by concomitant injection with PGE_2_ (14). Similarly, in women, inhibitors for PTG synthase resulted in the reduced ovulation rate (15, 16). Together, all this evidence points to crucial roles of these mediators in the ovulatory process across many species.

Previous studies have suggested cross regulation among P4/PGR, EGF-like factors, and PTGs in periovulatory follicles. For instance, knockout mice studies showed the positive cross regulation between EGF signaling and PTGs in periovulatory follicles (10, 17). Meanwhile, P4/PGR regulation on these mediators appeared to be time dependent and species specific (5, 17–19).

In humans, there are limited studies on the expression of these factors during the ovulatory period. A recent microarray analysis listed EGF-like factors (*AREG* and *EREG*) and PTG synthases (*PTGS2* and *PTGES*) as differentially upregulated genes in granulosa cells isolated at 36 hours post-hCG compared with those obtained before hCG administration (20). There are only two reports showing *PGR* expression in granulosa cells of dominant follicles collected during the LH surge (21, 22). Therefore, more comprehensive study is needed to clarify exactly when and where these three key ovulatory mediators are induced and whether they cross regulate in periovulatory follicles.

Based on previous data, we hypothesized that the LH surge increases P4/PGR, EGF signaling, and PTGs by coordinating the precisely timed cross regulation among these mediators in human periovulatory follicles. This study was performed (1) to determine the expression of the main components of these three key ovulatory mediators using timed human periovulatory follicles, (2) to establish an *in vitro* model that can mimic *in vivo* periovulatory changes in the expression of these mediators, and (3) to dissect the regulatory mechanism(s) by which LH/hCG coordinates the upregulation of these mediators in human periovulatory granulosa cells.

## Materials and Methods

### Materials

Unless otherwise noted, all chemicals and reagents were purchased from Sigma Chemical Company or Invitrogen Life Technologies, Inc. RU486 and Prostaglandin E_2_ and F_2_*_α_* enzyme immunoassay kits were purchased from Cayman Chemical.

### Human tissue collection: *in vivo* ovulatory follicles and granulosa cells

The protocol using human tissues is approved by the Human Ethics Committee of the Sahlgrenska Academy at the University of Gothenburg, and all patients gave their informed written consent before participating. All participants recruited had planned laparoscopic sterilization and had not taken hormonal contraceptives for at least 3 months prior to their enrollment as previously described (23). Women were also monitored by transvaginal ultrasound for two to three menstrual cycles before surgery to ascertain cycle regularity and to monitor the growth of a dominant follicle during the follicular phase. These patients were divided into four groups: pre-, early, late, and postovulatory phase. In the preovulatory group, surgery was performed when the follicle reached >14 mm and no more than 17.5 mm in diameter (mean, 15.7 ± 0.8 mm) prior to the endogenous LH surge. These patients were not given hCG. The remaining women were given 250 µg of recombinant hCG (Ovitrelle) and were divided into three groups: early ovulatory (surgery between 12 and 18 hours post-hCG), late ovulatory (surgery between 18 and 34 hours post-hCG), and postovulatory (between 44 and 70 hours post-hCG). To confirm these patients followed a normal hormonal pattern before the LH surge or after hCG administration, blood samples were taken at surgery and measured for serum progesterone and estradiol. The patient characteristics, including steroid levels, are shown in Supplemental Tables 1 and 2.

The whole intact follicle was removed using laparoscopic scissors and either processed for immunohistochemical analysis as subsequently described or placed on ice and brought to the laboratory for dissection. For gene expression analysis, the follicle was bisected with scissors, and mural granulosa cells were gently scraped off from the interior of the follicle by small tissue forceps. The follicular fluid and cell suspension were combined and centrifuged at 500 × *g* to pellet granulosa cells. The isolated granulosa cells were frozen at −70°C.

### Isolation and culture of human granulosa/lutein cells

Human granulosa cells were obtained from aspirates of *in vitro* fertilization (IVF) patients. The granulosa cell collection protocol was approved by the Institutional Review Board of the University of Kentucky Office of Research Integrity. Ovarian hyperstimulation was induced by the administration of recombinant human follicle-stimulating hormone in individualized doses to IVF patients at the Bluegrass Fertility Center (Lexington, Kentucky). IVF patients were then administered with hCG on days 9 to 11, and dominant follicles were aspirated 36 hours later. The experiments with human granulosa/lutein cells (hGLCs) were carried out as described previously (23). Immediately after retrieval of COCs, the remaining cells in aspirates were subjected to a Percoll gradient centrifugation to remove red blood cells. The isolated cells were resuspended with OptiMEM media supplemented with 10% fetal bovine serum and antibiotic-antimycotic (Invitrogen) and placed onto culture plates (2.5 × 10^5^ cells/mL). The cells were cultured at 37°C in 5% CO_2_ for 6 days, and media was changed every 24 hours. At the end of 6 days, the cells were treated with or without various regents ± hCG (1 IU/ml+L) in OptiMEM media supplemented with antibiotic-antimycotic and further cultured for stated hours.

### Immunohistochemical analysis

Follicles were fixed in 4% formaldehyde, embedded in paraffin, sectioned (7 μm), and processed for immunostaining. Briefly, heat-induced epitope retrieval was performed in a Decloaking Chamber (Biocare Medical) using a low pH Target Retrieval Solution (Dako). Primary antibody incubation was carried out at 4°C overnight for PGR (prediluted; Dako), PTGS2 (1:200; Cell Signaling Technology), and AREG (1:500; Sigma Chemical Company). The antibody was detected using an appropriate ImmPRESS-AP alkaline phosphatase kit (Vector Laboratories) and VECTOR Red AP chromogen (Vector Laboratories) according to manufacturer’s instructions. Slides were counterstained with hematoxylin. The negative control slides were prepared in an identical manner and processed without primary antibody.

### Analysis of gene expression

Total RNA was isolated from granulosa cells and whole follicles using a TRIzol Reagent or RNeasy Mini Kit according to manufacturer’s instructions. The synthesis of first-strand complementary DNA was performed by reverse transcription of 500 ng total RNA using superscript III with Oligo(dt)_20_ primer. The levels of messenger RNAs (mRNAs) for genes examined were measured by quantitative polymerase chain reaction (PCR) using TaqMan primers (*AREG*, *PTGS2*, and *PGR*) as described previously (23) or Brilliant 3 Ultra-Fast SYBR green according to the manufacturer’s protocol (Stratagene). Oligonucleotide primers corresponding to each gene were designed using Primer3 software and are listed in Supplemental Table 3. The specificity for each primer set was confirmed by both running the PCR products on a 2% agarose gel and analyzing the melting curve using the MxPro qPCR analysis program. The relative abundance of the target transcript was normalized to the endogenous reference gene, *GAPDH* or *RNA18S5*, and calculated according to the 2^-ΔΔCT^ method.

### Western blot analysis

Whole cell lysates were denatured by boiling for 5 minutes and separated by sodium dodecyl sulfate–polyacrylamide gel electrophoresis on a 10% polyacrylamide gel and then transferred onto a nitrocellulose membrane. The membrane was incubated overnight at 4°C in 1% casein solution containing primary antibodies against PGR, PTGS2 (Cell Signaling Technology), PTGES (Cayman Chemical), or *β* actin (Santa Cruz). The blots were incubated with the respective secondary horseradish peroxidase–conjugated antibody for 1 hour. Peroxidase activity was visualized using the SuperSignal West Pico Chemiluminescent Substrate (Pierce Chemical Co.).

### Hormone assay

The concentration of progesterone was measured using an Immulite kit as described previously (23). Assay sensitivity for the Immulite %was 0.02 ng/mL. The intra- and interassay coefficients of variation were 7 and 12%, respectively.

The concentrations of PGE_2_ and PGF_2_*_α_* were measured by enzyme immunoassay kits according to manufacturer’s instructions. The assay sensitivities for PGE_2_ and PGF_2_*_α_* were 8.7 and 3.0 pg/mL, respectively. The intra- and interassay coefficients of variation were 3.7% and 8.3%, respectively, in PGE_2_ and 9.0% and 9.1%, respectively, in PGF_2_*_α_*.

### Chromatin immunoprecipitation assay

Chromatin immunoprecipitation (ChIP) assay was performed on putative PGR binding sites in *PTGS2, PTGES*, and *SLCO2A1* promoter regions using a ChIP-IT kit (Active Motif). Briefly, hGLCs were fixed with Complete Cell Fixative Solution for 15 minutes and terminated by adding Stop solution. The cell pellet was resuspended in 5-mL ice-cold Swelling Buffer and sonicated in 1 ml ChIP Buffer (Active Motif) at a 5.0 power level for 20 minutes with 30-second sonication and 30-second intervals with a Fisher Sonic Dismembrator Model 550 (Fisher Scientific). Sheared chromatin was immunoprecipitated overnight at 4°C with anti-PGR IgG (3 µg/mL) or negative control IgG. The immunoprecipitated chromatin and input chromatin (1:10 dilution) were analyzed by PCR using the primers designed to amplify fragments of the PGR responsive element in respective promoter regions (Supplemental Table 2). Amplified PCR products were run on a 2% agarose gel, stained with ethidium bromide, and visualized under ultraviolet light.

### Statistical analyses

All data are presented as mean ± standard error of the mean. Data were tested for homogeneity of variance by the Levene test, and log transformations were performed, as appropriate. Student *t* test or one-way analysis of variance was used to test differences in levels of mRNA for each gene across time of tissue collection, time of culture, or among treatments *in vitro*. If analysis of variance revealed significant effects, the means were compared by Duncan test, with *P* < 0.05 considered significant.

## Results

### hCG increases the expression of PGR, PTGS2, and AREG in human periovulatory granulosa cells *in vivo*

**Figure 1.**
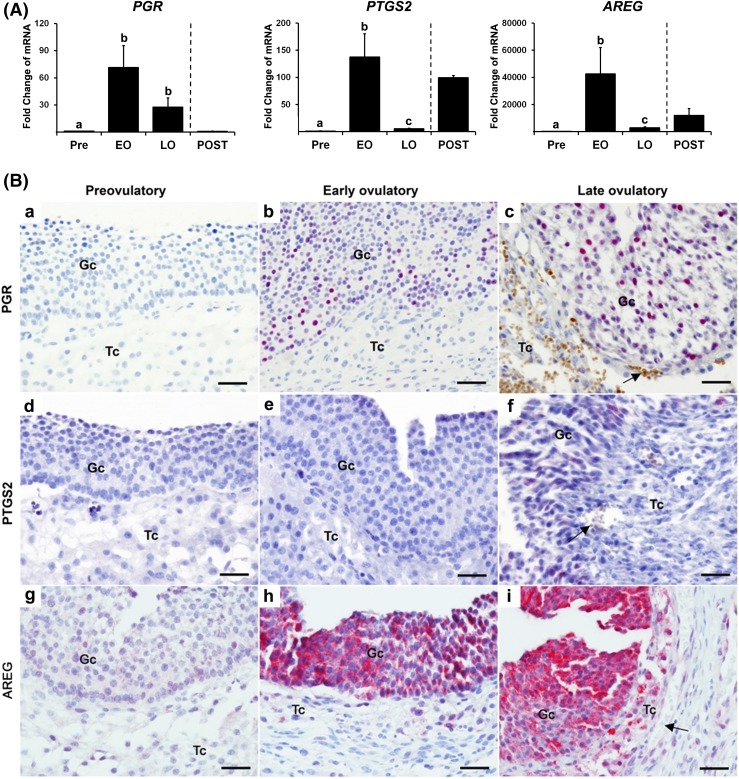
hCG increases the expression of *PGR*, *PTGS2*, and *AREG* in human periovulatory follicles. Dominant follicles were retrieved from the ovaries of women undergoing laparoscopic tubal sterilization before the LH surge or at defined hours after hCG administration and divided into four phases: pre- (Pre, n = 6), early (EO, n = 5), late (LO, n = 6), and post- (POST, n = 2) ovulatory phases as described in the Materials and Methods section. (A) The levels of mRNA for each transcript were measured by quantitative PCR in granulosa cells isolated from a dominant follicle collected at Pre, EO, and LO and whole follicles retrieved at POST and normalized to the levels of *GAPDH* mRNA in each sample. The levels of transcript were presented as fold change to Pre levels. Bars with no common superscripts are significantly different (*P* < 0.05). (B) Paraffin-embedded sections (7 µm) of dominant follicles were subjected to immunohistochemical analyses to detect PGR, PTGS2, and AREG. Pink/purple staining represents positive signals for PGR, PTGS2, and AREG protein. Arrows indicate red blood cells. Scale bar, 100 µm for all images. Gc, granulosa cells; Tc, theca cells.

The levels of *PGR* mRNA in granulosa cells were dramatically increased in the early ovulatory phase (~75-fold) compared with those obtained before hCG administration. The levels of *PGR* mRNA were maintained during the late ovulatory phase but decreased after ovulation to the level at the preovulatory stage [Fig. 1(A)]. Meanwhile, the levels of mRNA for *PTGS2* and *AREG* showed a triphasic pattern of change. The levels of mRNA for both genes peaked at the early ovulatory phase, rapidly declined by the late ovulatory phase, and then appeared to increase again after ovulation [Fig. 1(A)].

Positive staining for PGR, PTGS2, and AREG was detected in dominant follicles obtained only after hCG administration [Fig. 1(B)]. PGR protein began to localize to granulosa cells of early ovulatory follicles and became more intense in late ovulatory follicles [Fig. 1(Bb) and 1(Bc)]. PTGS2 protein was also detected in the granulosa cell layer of late ovulatory follicles [Fig. 1(Bf)]. Similarly, intensive immune-positive staining for AREG protein was localized to early and late ovulatory follicles; the staining is predominant in granulosa cells, but theca cells were also stained positively [Fig. 1(Bh) and 1(Bi)].

### hCG stimulates production of P4 and PTGs and expression of key ovulatory mediators *in vitro*

**Figure 2.**
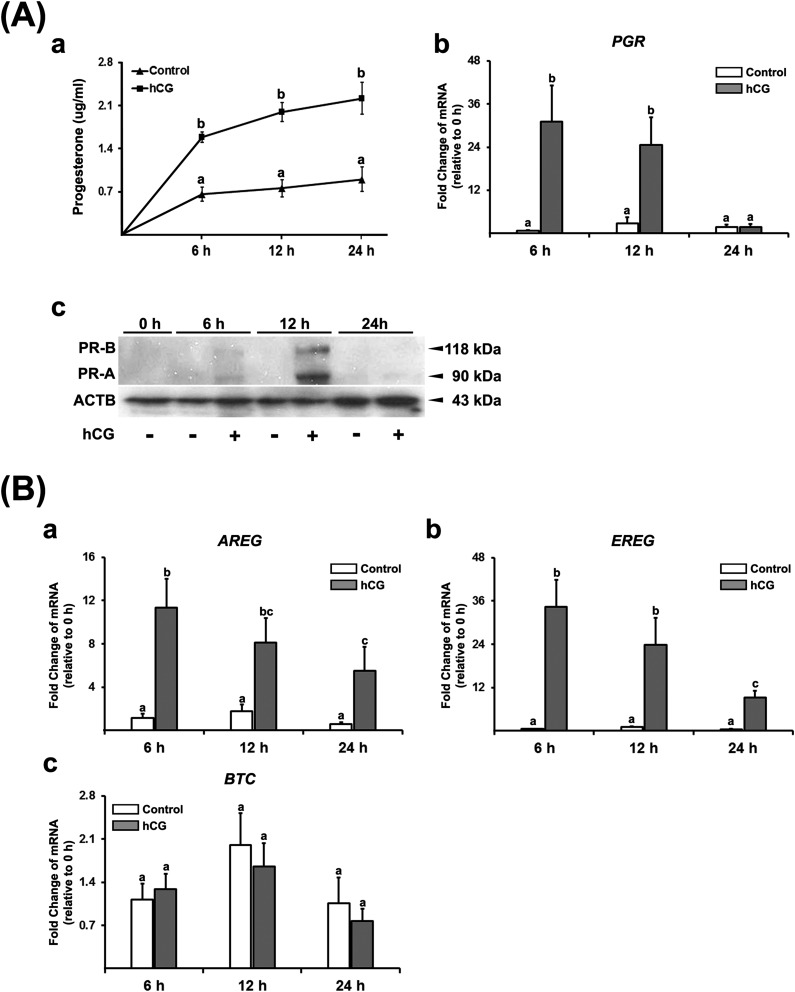
hCG increases progesterone production and the expression of *PGR* and EGF-like factors. Granulosa/lutein cells obtained from IVF patients were cultured for 6 days and then treated without (Control) or with hCG (1 IU/mL) for 6, 12, or 24 hours. (Aa) The concentration of P4 was measured in conditioned media (n = 5). (Ab) The levels of *PGR* mRNA were measured by quantitative PCR and normalized to the levels of *RNA18S5* in each sample (n = 4 independent experiments). Bars with no common superscripts are significantly different (*P* < 0.05). (Ac) Two isoforms of PGR protein (PR-A and PR-B) were detected by Western blots. Each lane (30 µg) was loaded with cell lysates. The membrane was reprobed with a monoclonal antibody against *β*-actin (ACTB) to assess the loading of protein in each lane. The experiments were repeated three times with independent samples. (B) The levels of (a) *AREG*, (b) *EREG*, and (c) *BTC* mRNA were measured by quantitative PCR and normalized to the levels of *RNA18S5* mRNA in each sample (n = 4 to 6 independent experiments for each time point). Bars with no common superscripts are significantly different (*P* < 0.05).

To determine whether the *in vivo* induction of local mediators during the periovulatory period can be recapitulated by hCG stimulation *in vitro*, we used hGLCs. The cells were acclimated in culture as described previously (23) and then treated with or without hCG (1 IU/mL). The levels of P4 in conditioned media were increased by hCG compared with controls [Fig. 2(Aa)]. hCG induced a transient increase in the levels of *PGR* mRNA; the levels peaked at 6 hours and then declined to basal levels by 24 hours [Fig. 2(Ab)], mimicking the *in vivo* expression pattern. hCG also induced PR-A and PR-B proteins: the levels were highest at 12 hours and reduced thereafter [Fig. 2(Ac)], indicating a time lag between the peak of mRNA and protein.

Next, we determined whether hCG promotes the production of EGF-like factors (AREG, EREG, and BTC) *in vitro*. hCG induced a transient increase in the levels of mRNA for *AREG* and *EREG*, with a peak level at 6 hours [Fig. 2(Ba) and 2(Bb)], similar to the *in vivo* expression pattern. In contrast, hCG had no effect on *BTC* mRNA [Fig. 2(Bc)].

**Figure 3.**
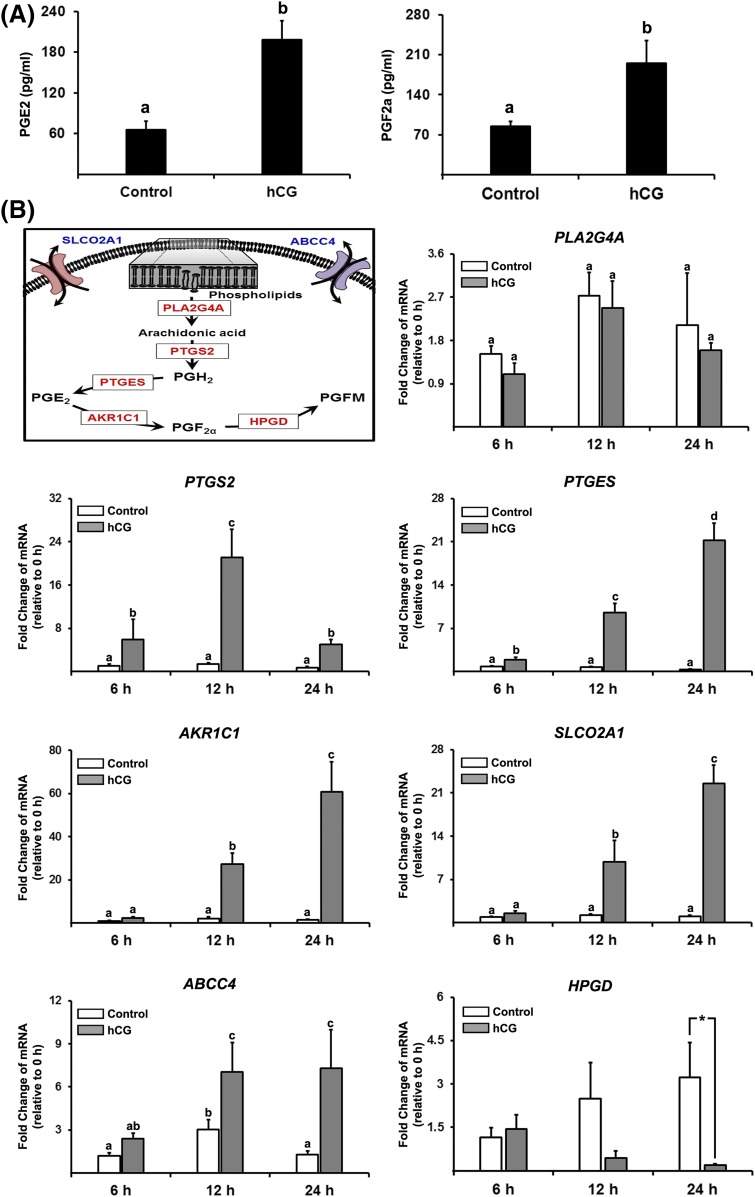
hCG increases PG production and the expression of PTG synthases and transporters. Primary hGLC were treated without (Control) or with hCG (1 IU/mL) for 6, 12, or 24 hours. (A) The concentrations of PGE_2_ and PGF_2a_ were measured in conditioned media (n = 5 independent experiments). (B) A schematic diagram of the PG biosynthesis pathway highlighting specific PTG synthases (*PLA2G4A*, *PTFS2*, *PTGES*, and *ARK1C1*), transporters (*SLCO2A1* and *ABCC4*), and metabolic enzyme (*HPGD*) involved. The levels of mRNA for these genes were measured by quantitative PCR and normalized to the levels of *RNA18S5* mRNA in each sample (n = 4 to 6 independent experiments). Bars with no common superscripts are significantly different (*P* < 0.05).

hCG increased the levels of PGE_2_ and PGF_2_*_α_* [Fig. 3(A)]. To dissect the mechanisms underlying these increases in PTGs, we measured the levels of mRNA for a series of PTG synthases and transporters known to be involved in PTG accumulation [Fig. 3(B)]. hCG increased the level of mRNA for all the PTG synthases and transporters examined, except for *PLA2G4A*. The hCG-induced increases in levels of *PTGS2* mRNA showed a biphasic pattern; the levels peaked at 12 hours and then decreased at 24 hours. Meanwhile, the levels of mRNA for PTG synthases, *PTGES* and *AKR1C1*, and a PTG transporter, *SLCO2A1*, were gradually increased by hCG, and their levels were highest at 24 hours. hCG also increased the levels of mRNA for *ABCC4*, but the increase was relatively moderate. Meanwhile, the levels of *HPGD* mRNA were decreased by hCG at 24 hours.

### Inhibition of P4/PGR and EGF signaling reduces P4 and PTGs production and the expression of PTG synthases and transporters and EGF-like factors

**Figure 4.**
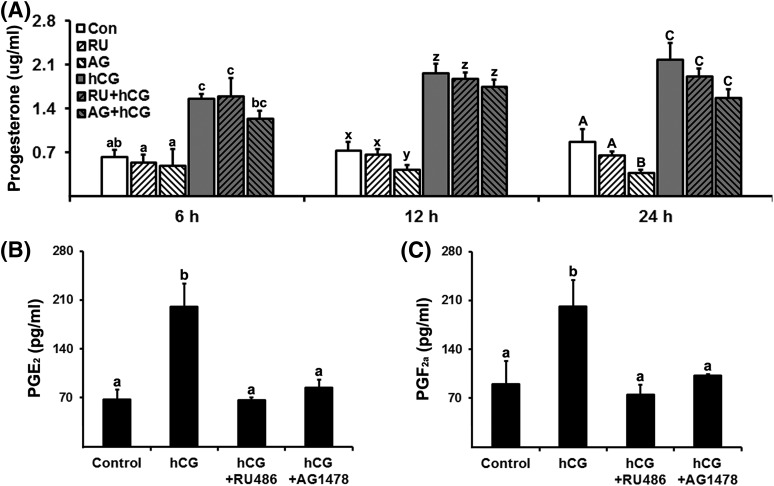
Inhibitors of P4/PGR action and EGFR signaling decreased progesterone and prostaglandin production. Primary hGLCs were treated with or without RU486 (progesterone receptor antagonist, 20 µM) or AG1478 (EGFR tyrosine kinase inhibitor, 10 µM) in the absence or presence of hCG (1 IU/mL) for 6, 12, or 24 hours. (A) The concentration of progesterone was measured in conditioned media. RU, RU486; AG, AG1478. Concentrations of (B) PGE_2_ and (C) PGF_2_*_α_* were measured in conditioned media collected at 24 hours after hCG treatment (n = 4 to 5 independent experiments). Bars with no common superscripts in each time point are significantly different (*P* < 0.05).

To determine whether P4/PGR and EGFR signaling are involved in P4 and PTG production, hGLCs were treated with PGR antagonist (RU486, 20 µM) or EGFR tyrosine kinase inhibitor (AG1478, 10 µM) in the absence or presence of hCG. The dosage of inhibitors was chosen based on previously published studies (19, 24, 25), and our pilot study showing a dose-dependent response (data not shown). RU486 had no effect on P4 levels both in control and hCG-stimulated cells [Fig. 4(A)]. AG1478 also had no effect on hCG-induced increases in P4 levels but decreased the basal production of P4 at 12 and 24 hours [Fig. 4(A)]. In contrast, hCG-induced increases in PGE_2_ and PGF_2_*_α_* were completely blocked by RU486 and AG1478 [Fig. 4(B) and 4(C)].

**Figure 5.**
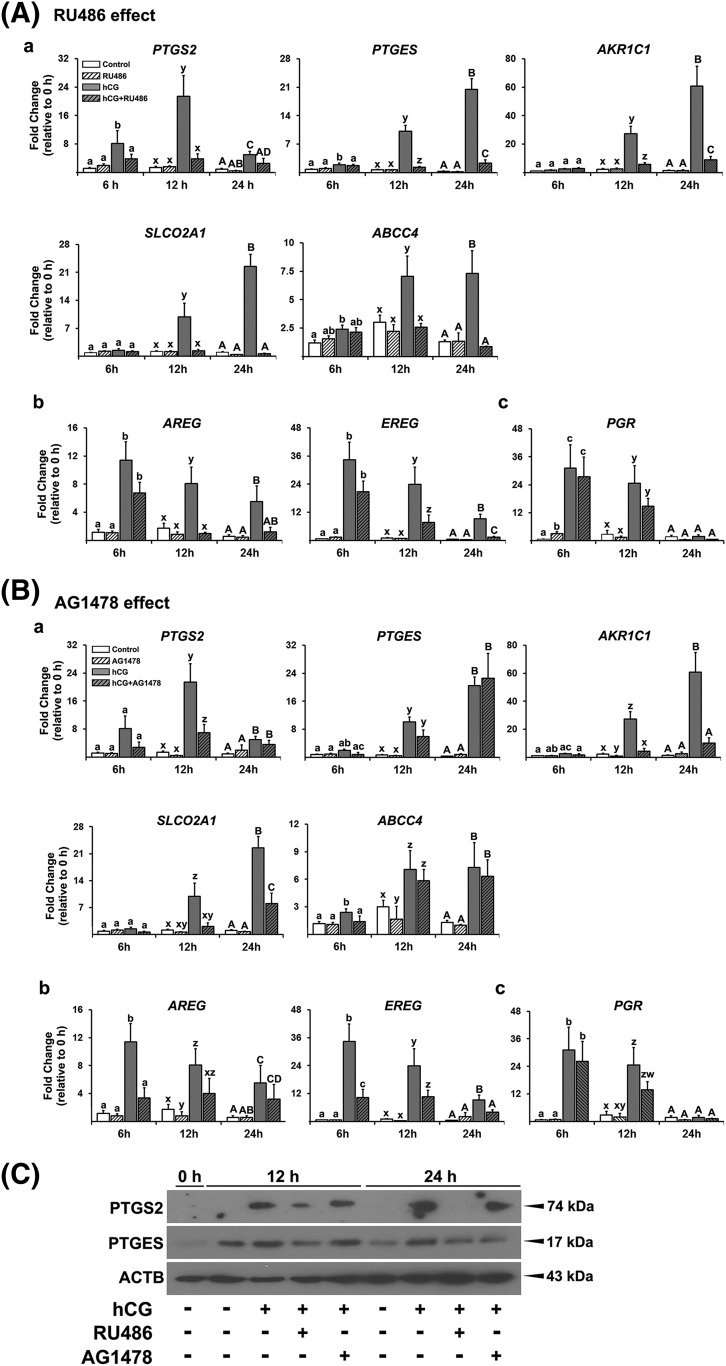
Inhibitors of P4/PGR action and EGF signaling decreased the levels of mRNA for PTG synthases and transporters and EGF-like factors. Primary hGLCs were treated with or without (A) RU486 (20 µM) or (B) AG1478 (10 µM) in the absence or presence of hCG (1 IU/mL) for 6, 12, or 24 hours. (A and B) The levels of mRNA for PTG synthases (*PTGS2*, *PTGES*, and *AKR1C1*), transporters (*SLCO2A1* and *ABCC4*), EGF-like factors (AREG and *EREG*), and *PGR* were measured by quantitative PCR. The levels of transcripts were normalized to those of *RNA18S5* mRNA in each sample (n = 4 to 6 independent experiments). Bars with no common superscripts in each time point are significantly different (*P* < 0.05). (C) Western blot analyses were performed with whole cell lysate (30 µg) to detect PTGS2 and PTGES protein. *β*-actin (ACTB) was used as a loading control. Experiments were repeated three times with independent samples.

To further determine how P4/PGR and EGF signaling regulate PTG accumulation, we assessed the impact of RU486 and AG1478 on the expression of PTG synthases and transporters. RU486 completely blocked the hCG-induced increase in mRNA levels for all PG synthases and transporters examined [Fig. 5(Aa)]. AG1478 also reduced hCG-induced increases in the levels of mRNA for *PTGS2*, *AKR1C1*, and *SLCO2A1*, but had no effect on the levels of mRNA for *PTGES* and *ABCC4* [Fig. 5(Ba)]. We also examined the effect of RU486 and AG1478 on the expression of *PGR* and EGF-like factors. RU486 had an inhibitory effect on hCG-induced increases in the levels of mRNA for *AREG* and *EREG* at 12 and 24 hours, but not at 6 hours [Fig. 5(Ab)]. AG1478 also reduced hCG-induced increases in the levels of mRNA for *AREG* and *EREG* [Fig. 5(Bb)] at 6 hours. Neither basal nor hCG-induced increases in *PGR* mRNA were affected by RU486 and AG1478 [Fig. 5(Ac) and 5(Bc)].

Consistent with the mRNA profile, PTGS2, and PTGES, protein was upregulated by hCG at 12 and 24 hours, and this hCG-induced induction was abolished by RU486 [Fig. 5(C)]. AG1478 reduced hCG-stimulated PTGES protein at 24 hours, but not at 12 hours [Fig. 5(C)].

### PGR silencing by small interfering RNA reduced the expression of PTG syntheses and transporter and PGR bound to promoter regions of PTGS2, PTGES, and SLCO2A1

**Figure 6.**
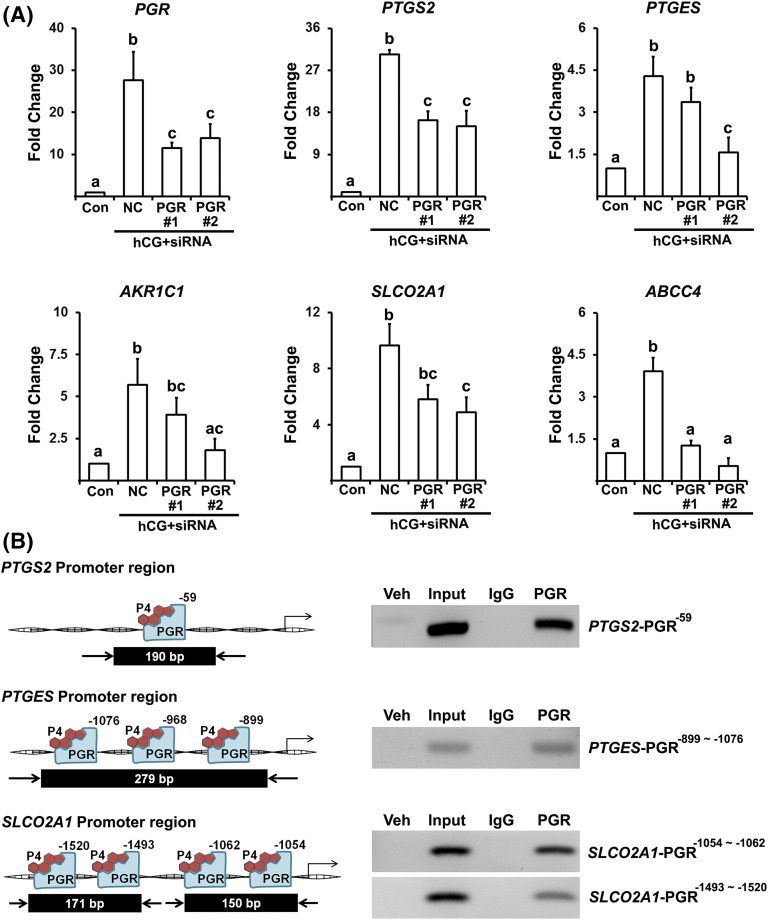
*PGR* siRNA reduced the expression of PTG synthases and transporters and PGR binds to promoter regions of *PTGS2*, *PTGES*, and *SLCO2A1*. (A) Primary hGLCs were treated with or without hCG (1 IU/mL) in the presence of negative control (NC) siRNA and *PGR* siRNA (#1 and #2) for 10 hours. The levels of mRNA for PTG synthases (*PTGS2*, *PTGES*, and *AKR1C1*), transporters (*SLCO2A1* and *ABCC4*) and *PGR* were measured by quantitative PCR. The levels of transcripts were normalized to *RNA18S5* mRNA in each sample (n = 3 independent experiments). Bars with no common superscripts in each time point are significantly different (*P* < 0.05). (B) ChIP assay was performed using hGLC treated with hCG (1 IU/mL) for 12 hours. DNAs immunoprecipitated with PGR antibody or IgG and input DNA were analyzed using primer pairs specific to promoter regions of the *PTGS2*, *PTGES*, and *SLCO2A1* gene and represented as arrows. Amplified DNA fragments containing PGR responsive elements are represented as a black box with the indicated PCR product size. Experiments were repeated three times, each with independent cultured cells.

RU486 acts as an antagonist for both PGR and glucocorticoid receptor. To verify that the impact of RU486 is through PGR, *PGR* small interfering RNA (siRNA) siRNA was used to reduce hCG-induced *PGR* expression [Fig. 6(A)]. *PGR* siRNA treatment resulted in reduced levels of mRNA for all PTG synthases and transporters examined [Fig. 6(A)]. To further assess how P4/PGR regulates the transcription of *PTGS2*, *PTGES*, and *SLCO2A1* in human granulosa cells, we screened for potential PGR binding sites in the 5′-flanking region of human *PTGS2*, *PTGES*, and *SLCO2A1* using a TFSEARCH and PROMO (Supplemental Fig. 1). ChIP data revealed enriched chromatin fragments containing the promoter regions of *PTGS2*, *PTGES*, and *SLCO2A1* [Fig. 6(B)] in the hGLC cultured with hCG for 12 hours.

## Discussion

This study revealed a rapid and precisely timed induction of key ovulatory mediators, *PGR*, *AREG*, and *PTGS2*, in granulosa cells of human periovulatory follicles. These findings are consistent with a previous study (20) that identified *AREG* and *PTGS2* as differentially upregulated genes in microarray data comparing the transcriptome between human granulosa cells isolated before the LH surge and 36 hours after hCG administration. However, in that study, *PGR* was not listed as differentially regulated. This discrepancy could be in part because of dynamic and rapid changes of these gene expressions. Specifically, *PGR*, *AREG*, and *PTGS2* mRNA levels were all rapidly increased within 12 hours after hCG administration. However, their profiles during the late and postovulatory period diverge; *PGR* mRNA levels declined after ovulation, whereas the levels of *AREG* and *PTGS2* mRNA were sharply decreased during the late ovulatory period, but then increased again after ovulation. By comparing a single time point (36 hours post-hCG), dynamic changes in transcript levels of these genes may be missed during the ovulatory period in the previous study. Together with our data documenting the upregulated expression of PGR, AREG, and PTGS2 protein in ovulatory follicles, the current study suggests that these three factors play an important role in the ovulatory process. In addition, the second rise of *AREG* and *PTGS2* mRNA in postovulatory follicles suggests the possible role of these two factors in corpus luteum formation/function in humans.

The present data from the *in vivo* study also revealed that the transcriptional regulation of these genes is species specific during the ovulatory period. For instance, in rodents, cattle, and monkeys, the LH surge/hCG-induced increase in levels of *Pgr* mRNA was short-lived, sharply declining even before ovulation (5, 26–28), whereas the level of *PGR* mRNA in humans declined only after ovulation. Species differences also exist in the expression profile of *PTGS2* and *AREG* mRNA. In rodents, the periovulatory upregulation of *Ptgs2* and *Areg* is transient and completely diminished in postovulatory ovaries (8, 29). However, in monkey ovaries, the levels of *PTGS2* and *AREG* mRNA were increased within 12 hours and remained elevated until 36 hours post-hCG (30, 31). In cattle, the level of *PTGS2* mRNA was increased during the late ovulatory period (32), whereas the induction of *AREG* mRNA was transient, highest at 6 hours post-hCG during the early ovulatory period (9). At present, there is no clear answer for how and why the expression pattern of these genes is diverse among different species. However, the understanding of the regulatory mechanisms by which the LH surge/hCG increases these genes expression would be the first step toward answering these questions.

To accomplish this, it is critical to establish an *in vitro* model that can mimic LH-/hCG-induced cellular changes *in vivo* in human preovulatory granulosa cells. Previously, we have shown that hGLC from IVF patients can respond to hCG after preincubation for 6 days (23). Indeed, these cells increased the production of P4 and PGE_2_/F_2a_ in response to hCG in this study. hCG also increased the expression of *PGR*, EGF-like peptides, and genes known to be involved in PG synthesis and transport. The importance of this finding is that this is an *in vitro* model where hCG increases all three key ovulatory genes/mediators in a time-dependent manner, similar to that observed *in vivo* (20), presenting this model as a useful tool that can be used to investigate the regulation and interaction of these mediators during the ovulatory period.

Using this model, we discovered that P4/PGR and EGF signaling coordinate hCG-stimulated PTG production in hGCL. For instance, studies using RU486 or *PGR* silencing showed that P4/PGR is required for hCG-induced increases in *PTGS2*, *PTGES*, *AKR1C1*, *SLCO2A1*, and *ABCC4* expression. Together with the evidence showing the direct binding of PGR on *PTGS2*, *PTGES*, and *SLCO2A1* genes, these data provided compelling evidence that hCG increases P4/PGR, which in turn upregulates the transcription of genes involved in PG synthesis and transport. Consistent with our data, RU486 inhibited LH-/hCG-induced increases in *PTGS2* expression and PTG production in bovine and human granulosa cell cultures (18, 19). However, *Pgr* null mice showed no difference in the expression of *Ptgs2* (5), indicating that the regulation of P4/PGR on PG production is species specific. Besides P4/PGR, EGF signaling was found to regulate the expression of *PTGS2*, *AKR1C1*, and *SLCO2A1*, but not *PTGES* and *ABCC4* in hGLC. In agreement with our findings, mutant mice with compromised EGF signaling showed reduced levels of *Ptgs2* mRNA (10). In a human granulosa cell line (SOVG), EGF-like factors increased the levels of *PTGS* mRNA (33). These data suggest that the coordinated regulation of P4/PGR and EGF signaling is required for the LH surge/hCG-induced increases in PTG production in human ovulatory follicles.

This study also unraveled the involvement of P4/PGR and EGFR activation in the regulation of *AREG* and *EREG* expression. Interestingly, AG1478 and RU486 inhibited *AREG* and *EREG* expression at 6 and 12 hours, respectively, suggesting the time-dependent regulation. Similarly, *Pgr* knockout mice showed reduced expression of *Areg* and *Ereg* at 8 hours post-hCG, and *Ereg* knockout mice showed reduced *Areg* expression in periovulatory ovaries (17, 34). Together, these data indicated that P4/PGR is involved in the maintenance of *AREG* and *EREG* expression, whereas EGF signaling facilitates the rapid, initial upregulation of its own ligands.

The rise in P4 production after the LH surge is crucial for ovulation and subsequent luteal formation and function. Previous studies have implicated a positive feedback regulation of P4 in hgLCs (24, 35). However, in our *in vitro* model, both RU486 and AG1479 showed no effect on hCG-stimulated P4 production. Similarly, Hirata *et al.* (36) showed that PGR antagonist (ZK98299, 100 μM) had no effect on P4 in a similar culture model, suggesting that P4/PGR may not regulate P4 production during the early ovulatory period. In contrast with our data, a recent study has shown that AG1479 (10 µM) inhibited hCG-stimulated P4 production in hGLCs (25). This study used slightly different cell and culture preparation methodology compared with ours. Therefore, further studies will be needed to clarify the involvement of EGF signaling in P4 production in human periovulatory follicles.

In summary, using a valuable *in vivo* model, this study documented for the precisely timed upregulation of *PGR*, *PTGS2*, and *AREG* in human periovulatory follicles. Moreover, we established a unique *in vitro* model that mimicked the *in vivo* hCG-induced upregulation of key ovulatory mediators. Our *in vitro* studies further demonstrated that P4/PGR and EGF signaling are not only necessary for hCG-induced increases in PG production by upregulating the expression of specific components of PTG synthases and transporters, but also potentiating EGF signaling by increasing the expression of EGF-like factors. Progestins and RU486 have been used as emergency contraceptives. The blockade of PG production has been explored as an alternative option for nonsteroidal contraceptive medicine. EGFs are known to induce oocyte maturation *in vitro* (37) and to improve oocyte competence (38). Therefore, our current findings of coordinated regulation among these mediators provide key information that can be translated directly to women’s health in terms of developing more effective contraceptives and applying this knowledge to design better strategies to improve fertility.
