# Supplementary material for: Coordinated Regulation Among Progesterone, Prostaglandins, and EGF-Like Factors in Human Ovulatory Follicles
Source: J Clin Endocrinol Metab. 2017 Mar 9;102(6):1971–82. doi: 10.1210/jc.2016-3153 (PMC5470773; doi:10.1210/jc.2016-3153)
Supplement: Supplementary file 1 [file jc.2016-3153.sm1.docx]

**Supplemental table 1: Characteristics for follicle size, time of surgery in relation to hCG, preoperative hormone values of peripheral blood (serum), menstrual cycle day of surgery, menstrual cycle length and age of the study population. All data is given as means ± SEM.**

|  | **Preovulatory**  **phase** | **Early ovulatory phase** | **Late ovulatory phase** | **Postovulatory**  **phase** |
| --- | --- | --- | --- | --- |
| **Follicle size (mm)** | **15.9 ± 0.7**  **range 14.0 – 17.0** | **17.1 ± 0.8**  **range 15.0 – 19.0** | **17.2 ± 0.8**  **range 15.0 – 20.0** | **16.7 ± 0.5**  **range 15.0 – 18.0** |
| **Hours after rhCG** |  | **14.0 ± 0.9**  **range 12.0 – 18.0** | **21.9 ± 0.8**  **range 19.0 – 24.0** | **49.1 ± 0.6**  **range 44.0 – 70.0** |
| **Progesterone (nmol/L)** | **0.4 ± 0.04**  **range 0.3 – 0.5** | - 1. **± 0.22**   **range 0.5 – 1.9** | **2.1 ± 0.3**  **range 1.5 – 3.0** | **3.1 ± 1.5**  **range 1.3 – 9.5** |
| **Estradiol  (nmol/L)** | **0.5 ± 0.1**  **range 0.2 – 1.0** | **0.6 ± 0.1**  **range 0.4 – 0.9** | **0.7 ± 0.1**  **range 0.6 – 0.9** | **0.3 ± 0.1**  **range 0.21 – 0.6** |
| **Cycle day of surgery** | **14.0 ± 0.3**  **range 13 – 15** | **12.6 ± 0.5**  **range 11 – 14** | **13 ± 0.6**  **range 11 – 15** | **13.1 ± 0.6**  **range 11 – 15** |
| **Cycle length**  **(days)** | **29.0 ± 0.6**  **range 28-35** | **27.2 ± 0.5**  **range 26 – 28** | **29.4 ± 0.9**  **range 28 – 32** | **28.4 ± 0.5**  **range 27-30** |
| **Age**  **(years)** | **35.4 ± 1.2**  **range 31 – 38** | **33.8 ± 1.4**  **range 30 – 38** | **37.4 ± 1.4**  **range 36 – 38** | **34.2 ± 0.5**  **range 33 – 36** |

All the patients recruited had a planned laparoscopic sterilization as a means of contraception and participated in the study voluntarily. The average parity of these patients is 2.24 (range 0 – 4, 0 for 2 patients).

**Supplemental table 2: Characteristics of IVF patients from whom granulosa cells were collected and used for RNA experiments in the present study [age, ethnicity, Body Mass Index (BMI), estradiol (E2), number (#) of antral follicle counted prior to surgery by transvaginal ultrasound, number of eggs retrieved, and diagnosis of individual patients.**

| **Patient** | **Age** | **Ethnicity** | **BMI** | **E2_D0** | **E2_D4** | **E2_D7** | **# of Follicle** | **# of Eggs**  **Retrieved** | **Diagnosis** |
| --- | --- | --- | --- | --- | --- | --- | --- | --- | --- |
| 1 | 24 | White | 26.4 | 90 | 580 | 2032 | 15 | 15 | male factor |
| 2 | 33 | Asian | 20.6 | 34 | 156 | 903 | 13 | 11 | unexplained |
| 3 | 36 | White | 37.2 | 79 | 570 | 1028 | 13 | 5 | anovulation |
| 4 | 37 | White | 41.6 | 69 | 213 | 785 | 10 | 8 | male factor |
| 5 | 31 | White | 20.0 | 21 | 201 | 630 | 15 | 27 | tubal factor |
| 6 | 31 | White | 39.4 | 58 | 181 | 357 | 8 | 6 | PCOS |
| 7 | 25 | White | 25.2 | 51 | 697 | 1554 | 18 | 36 | male factor |
| 8 | 36 | Asian | 20.9 | 71 | 699 | 1939 | 10 | 7 | male factor |
| 9 | 28 | Black | 27.5 | 82 | 953 | 1850 | 18 | 18 | unexplained |
| 10 | 34 | White | 20.7 | 45 | 1407 | 3540 | 20 | 25 | tubal factor |
| 11 | 27 | White | 22.1 | 20 | 439 | 1145 | 15 | 22 | donor |

The E2_0 is the value measured after the start of the period induced by oral contraceptives and before any FSH medications. The E2_D4 and E2_D7 are the measurement after 4 and 7 days of FSH administration, respectively. GnRH antagonist is begun the evening of day 4, 5 or 6 depending on when the lead follicle reaches a mean diameter of 12 mm.

**Supplemental table 3: Primer sequences (Forward and Reverse) used for real-time PCR analyses and ChIP assays**

| **Genes** | **Accession no** | | **Forward primers** | **Reverse primers** |
| --- | --- | --- | --- | --- |
| **For Real-time PCR** | | |  |  |
| *PGR* | | NM_001202474.3 | ATCAACTAGGCGAGAGGCAA | TGCCACATGGTAAGGCATAA |
| *PLA2G4A* | | NM_024420.2 | AACCAAGTGCCTGTGGTAGC | GGCCCTTTCTCTGGAAAATC |
| *PTGS2* | | NM_000963.3 | TGAAACCCACTCCAAACACA | GAGAAGGCTTCCCAGCTTTT |
| *PTGES* | | NM_004878.4 | GCTGATCACACCCACAGTTG | CCAGGAAAAGGAAGGGGTAG |
| *AKR1C1* | | NM_001353.5 | ATTCCCATCGACCAGAGTTG | CATGTGGCACAGAGATCCAC |
| *SLCO2A1* | | NM_005630.2 | TCAACATGAGCTCTGCAACC | ATCAACAAGAACTGCACCCC |
| *ABCC4* | | NM_005845.4 | GGCTTGTGCTCTGAAAAAGG | CTGAGAGGATCGTCCAGGAG |
| *HPGD* | | NM_000860.5 | AAAACCAATTTTGCCACAGC | GAGTTTTACCCAAAGCAGGTG |
| *AREG* | | NM_001657.3 | CGAACCACAAATACCTGGCT | TGCATGTTACTGCTTCCAGG |
| *EREG* | | NM_001432.2 | CGTGTGGCTCAAGTGTCAAT | AGTGTTCACATCGGACACCA |
| *BTC* | | NM_001316963.1 | TGGGAATTCCACCAGAAGTC | GTTTCCGAAGAGGGACACAG |
| *RNA18S5* | | NR_046235.1 | GTAACCCGTTGAACCCCATT | CCATCCAATCGGTAGTAGGG |
| **For ChIP assay** | | |  |  |
| *PTGS2* | | NC_000001.11 | CTGGGTTTCCGATTTTCTCA | ATTGGTGCCTAACCGAGAGA |
| *PTGES* | | NC_000009.12 | CACCCAAGATCAGACCCAAC | TTCCAGGCAAATCCTCAAAC |
| *SLCO2A1 (1)* | | NC_000003.12 | GGATGTACCTCGCTCTGTGG | ATACCTTTCTGGGCCTGGTC |
| *SLCO2A1 (2)* | |  | TTCTGGTCCCTGTTTGGTTC | GCAGTCGGCTAAGAAAGCTG |


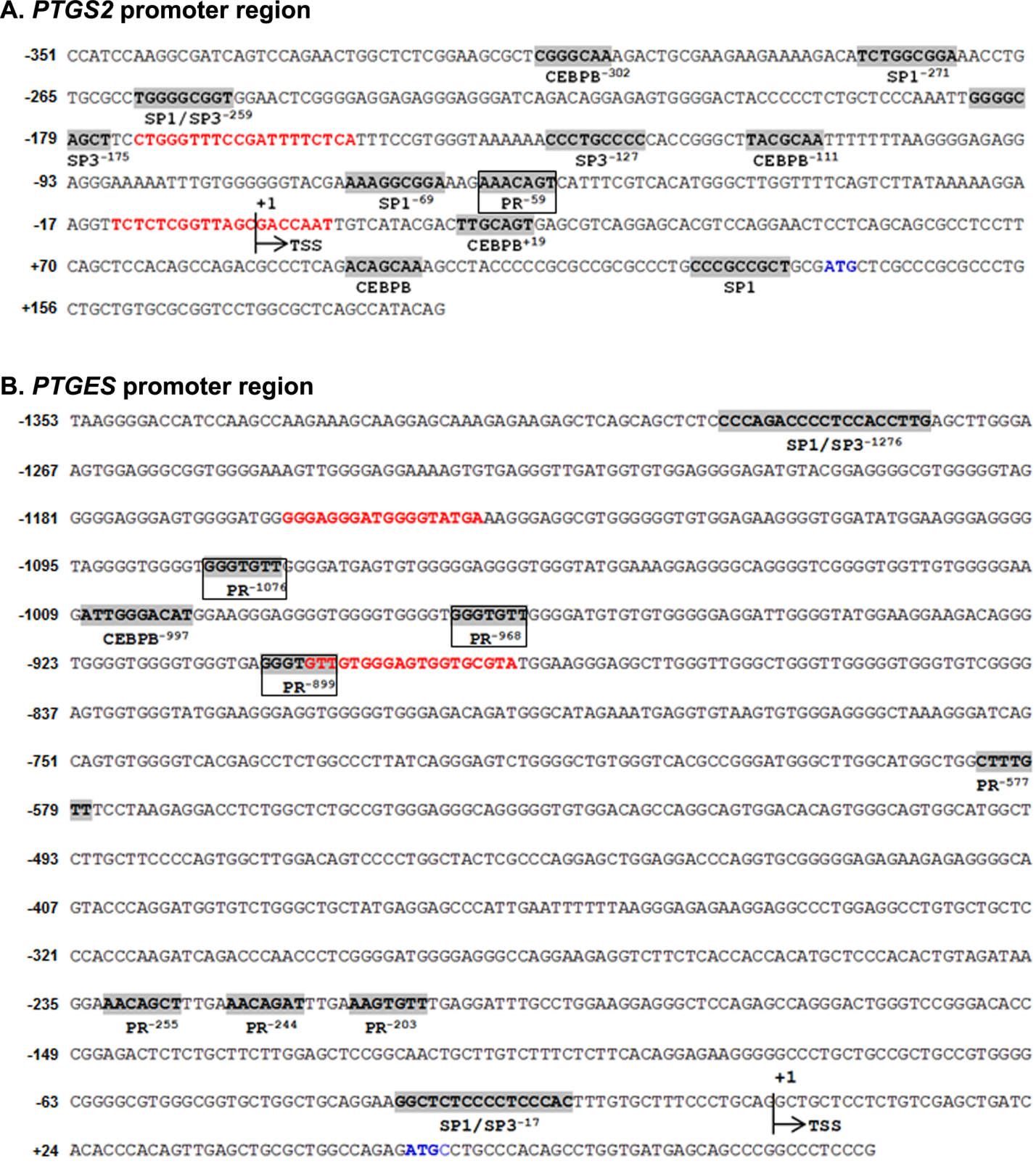


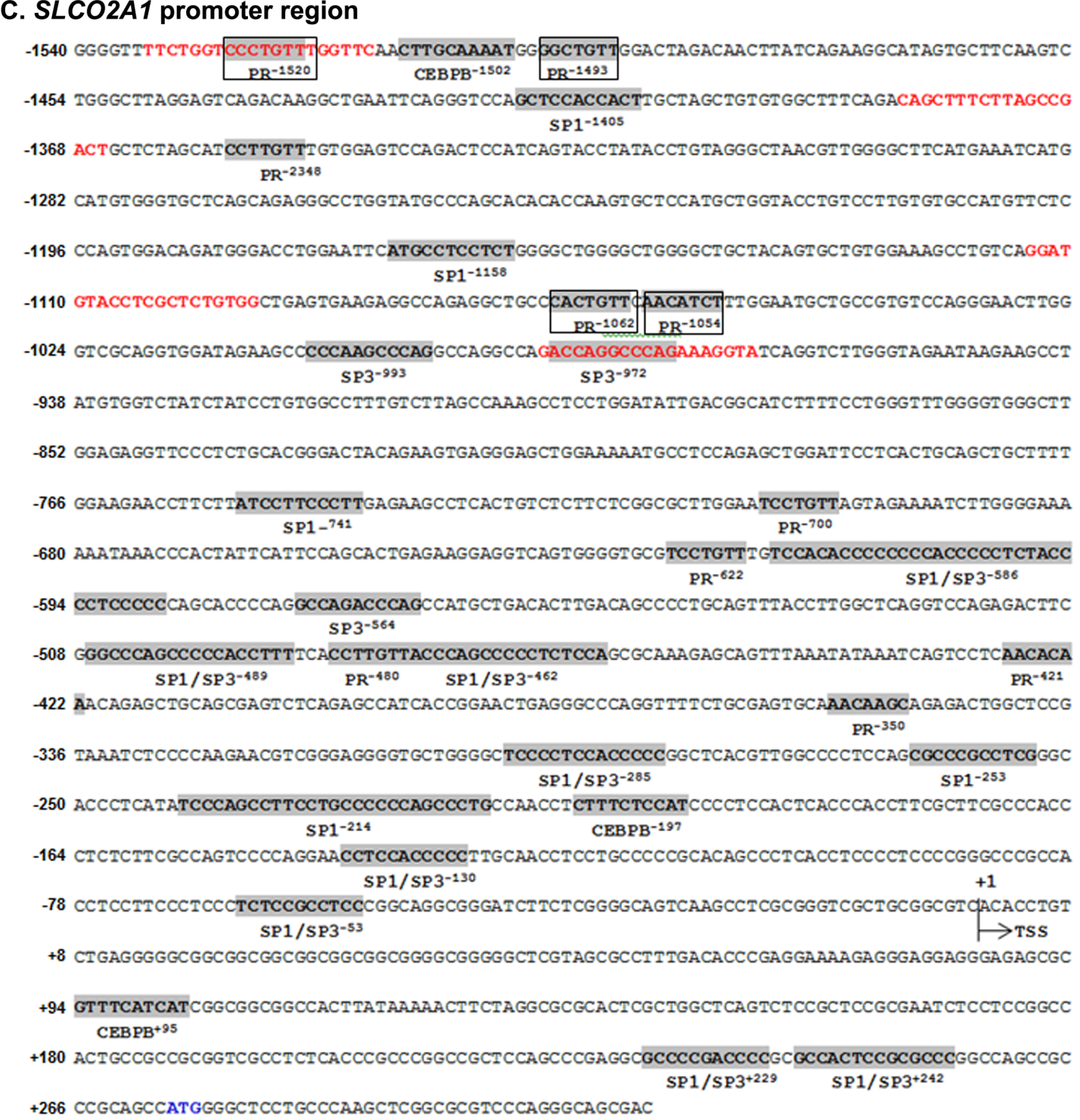


**Supplemental Fig. 1: Transcription factor binding motifs in nucleotide sequences of promoter regions for human *PTGS2*, *PTGES*, and *SLCO2A1***

Promoter sequences of human *PTGS2* (A), *PTGES* (B), and *SLCO2A1* (C) were analyzed using a genomic library. Nucleotide sequences are numbered from TSS at +1. The sequences of the oligonucleotide primers are colored in red. Putative transcription factor binding sites (highlighted in gray) are predicted by TFSEARCH.
